# The impact of boarding schools on the development of cognitive and non-cognitive abilities in adolescents

**DOI:** 10.1186/s12889-023-16748-8

**Published:** 2023-09-23

**Authors:** Fang Chang, Yanan Huo, Songyan Zhang, Hang Zeng, Bin Tang

**Affiliations:** Center of Experimental Economics in Education, Shaanxi Normal University Shaanxi Province, No. 620 West Chang’an Street, Chang’an District, Xi’an, 710119 China

**Keywords:** Boarding, Cognitive ability, Non-cognitive ability

## Abstract

**Background:**

Since China adopted a policy to eliminate rural learning centers, boarding has become an important feature of the current rural student community. However, there is a lack of consensus on the impact of boarding schools on students' cognitive and non-cognitive development. This study investigates the effect of boarding schools on the development of cognitive and non-cognitive abilities of junior high school students in rural northwest China.

**Methods:**

Using a sample of 5,660 seventh-grade students from 160 rural junior high schools across 19 counties, we identify a causal relationship between boarding and student abilities with the instrumental variables (IV) approach.

**Results:**

The results suggest that boarding positively influences memory and attention, while it has no significant effect on other cognitive abilities such as reasoning, transcription speed, and accuracy. Furthermore, we find no significant association between boarding and the development of non-cognitive skills.

**Conclusions:**

Given the widespread prevalence of boarding schools in rural regions, our study highlights the growing importance of improving school management to promote the development of students’ cognitive abilities and integrating the development of non-cognitive or social-emotional abilities into students’ daily routines.

## Introduction

Human ability is composed of cognitive and non-cognitive components, both of which are crucial to an individual's life [1, 2]. The influence of cognitive and non-cognitive abilities has been observed in various aspects of life, including academic performance, educational choices, wages, labor market outcomes, employment decisions, health behaviors, and social integration [3–5].

Cognitive and non-cognitive abilities are core components of human capital [1, 2, 6]. Cognitive abilities are the endowments for extracting, storing and utilizing information from the objective world, This encompasses skills such as logical reasoning, abstract thinking and memory [7], while non-cognitive abilities have emerged as a concept distinct from cognitive abilities, aiming to distinguish factors beyond cognitive itself. These encompass qualities such as motivation, authority, work norms, self-control, perseverance, and more [8]. Numerous researches have shown that cognitive and non-cognitive abilities play an important role in academic performance, educational decisions, wages, labor market performance, employment choices, health behaviors and social integration [1, 3–5, 8, 9]. Prior research has revealed a considerable disparity between the cognitive abilities of rural and urban students, with urban students scoring significantly higher on word and mathematics tests by 0.75 and 0.54 standard deviations, respectively [6, 10]. Rural students also tend to exhibit lower levels of non-cognitive skills, including depression, self-esteem, and values, with left-behind children experiencing even greater disadvantages [11].

Beginning in 2001, China adopted a policy to eliminate rural learning centers, leading to the consolidation of educational resources and the growth of rural boarding schools. By the end of 2016, 26.08 million rural students were enrolled as compulsory boarders, comprising 27.5% of the total student population. Of these, 16.66 million were boarding students in rural junior high schools, amounting to a boarding rate of 58.6% [12]. Therefore, a comprehensive evaluation of the cognitive and non-cognitive development of boarding students in rural areas has become essential.

Studies have shown that the communal learning environment in boarding schools can increase learning time, optimize teaching resources, and provide more opportunities for boarders to communicate with their teachers and peers [13, 14]. However, boarding students may also be exposed to at-risk peers, which can have negative effects on their development [15, 16]. Boarding can also cause stress for students as they are separated from their familiar surroundings and parents, which can be particularly significant during critical growth stages [17]. Consequently, there is a lack of consensus on the impact of boarding schools on students' cognitive and non-cognitive development.

Extant research on boarding schools has primarily focused on elite schools in developed countries, which have generally been associated with positive academic performance [18]. However, public boarding schools have been set up in many developed countries for marginalized groups, such as the SEED public boarding schools in the US and the Internet Excellence program in France. Quasi-experimental studies have shown that boarding has had a significant positive impact on the academic performance of disadvantaged students in reading and mathematics [19]. Similarly, rural boarding schools in France have positively impacted academic performance, particularly for outstanding boarders, with significant improvements in French and mathematics scores two years after enrollment [14]. Nonetheless, studies in Turkey have reported a negative correlation between boarding and academic performance in Grades 5 to 9 [15]. Boarding also has a significantly negative impact on students' mental health, with boarders displaying more problem behaviors, such as anxiety, depression, hostility, substance abuse, alcohol dependency, and school bullying [20, 21]. Notably, the impact of boarding varies at different stages of development. For instance, Mander et al. (2015) found no significant differences in social, emotional, and psychological well-being between boarders and non-boarders in elementary schools [22]. However, boarders in secondary schools exhibited a higher incidence of emotional difficulties, depression, anxiety, and stress compared to non-boarders. Given the mixed evidence, it is crucial to carefully consider the potential positive and negative impacts of boarding, especially for disadvantaged students attending public boarding schools.

As boarding school enrollment continues to rise in China, researchers have investigated the effects of boarding on students' cognitive and non-cognitive abilities and reported conflicting findings. Qiao and Di (2014) found that boarding significantly improved rural students' performance in mathematics [23], while Mo et al. (2012) reported a significant negative effect of boarding on primary school students' math scores [24]. Similarly, Wang et al. (2016), Li et al. (2018), and Zhu et al. (2019) found that boarding had no significant impact on students' standardized math scores or even reduced their standardized language scores [25–27]. Most studies indicate that boarding has a negative impact on students' non-cognitive skills. Rural boarders are more likely to experience bullying, loneliness, and depression in schools and have lower self-esteem, resilience, and emotional intelligence than non-boarders [27–30]. Taken together, these results suggest that the effects of boarding on students' academic and non-academic outcomes are complex and may vary depending on a range of factors, including the type of boarding school, the students' developmental stage, and their socio-economic background.

This paper aims to contribute to the existing literature on the impact of boarding on rural students' cognitive and non-cognitive abilities in three ways. Firstly, the literature has primarily measured cognitive abilities using subject-specific scores, which may not fully capture the breadth of cognitive abilities. There are numerous studies on cognitive abilities in different disciplines. psychologists commonly differentiate between fluid intelligence, which emphasizes more general capacities such as logical reasoning and abstract thinking, and crystallized intelligence, which is related to the accumulation of concrete knowledge and experience [31, 32]. Academic performance, such as math and reading tests is often used to measure crystallized intelligence [33]. Conversely, fluid intelligence is frequently assessed through quotient tests (IQ tests), exemplified bu tools like the WISC-IV and Raven's Standard Progressive Matrices [34]. To improve accuracy and precision in measuring cognitive abilities, this paper utilizes the Wechsler Intelligence Scale for Children (WISC), Raven's Standard Progressive Matrices, and standardized mathematics scores. Secondly, the literature has relied on self-administered questionnaires to measure non-cognitive abilities, which may lack comprehensiveness and comparability. In contrast, this paper uses the Big Five Personality Inventory to measure non-cognitive abilities accurately [35]. Finally, prior studies have examined the effects of boarding on cognitive or non-cognitive abilities separately, which prevents a comprehensive assessment of the impact of boarding on students' human capital.

This study uses an instrumental variable approach to address endogeneity issues and analyzes data from 160 junior high schools in rural northwest China to illustrate the effects of boarding on students' cognitive and non-cognitive abilities. The results indicate a significant positive effect of boarding on the cognitive abilities of rural junior high school students, particularly in memory and attention, areas associated with fluid intelligence. However, boarding has no significant impact on the non-cognitive abilities of rural students. Furthermore, we provide evidence of heterogeneity in the impact of boarding on cognitive and non-cognitive abilities by gender. We also find a significant positive effect on the cognitive abilities of left-behind children and students from families with better socioeconomic status.

## Method

### Participants

We conducted our study on first-year rural high school (seventh grade) students in three prefectures from two provinces in northwest China. These provinces were below the national median in terms of GDP, according to the National Bureau of Statistics of China (2015). Hence, the sample of rural students in these provinces can be considered representative of students in low-income areas in rural China.

We constructed our sample in two steps. First, we selected 23 counties from three prefectures, two counties with more developed economic status were excluded, and the remaining were included. Second, we obtained a list of all 496 junior high schools from the counties in Step 1. After excluding non-rural schools and schools with less than 20 students in the seventh grade (to address potential sample attrition or school merger issues), we obtained a final sample containing 5,660 seventh-grade students from 160 schools (see Table 1).

**Table 1:** Sample distribution

	**Counties**	**Schools**	**Students**	**Boarding students**	**Non-boarding students**	**Students tested by Raven**	**Students tested by Wechsler**	**Students tested by the Big five**
Total Sample	19	160	5660	2601	3059	2503	472	5166
Province A	7	90	3413	841	2572	1530	208	3136
Province B	12	70	2247	1760	487	973	264	2030

### Procedure

The sample was collected in two phases. The first phase was carried out in 2015, which involved administering tests to collect information on basic details of the sample students, mathematics teachers, and schools using questionnaires. Mathematics scores of students were also collected through tests (see Table 2). In the second phase, conducted in 2016, additional tests were administered, which included more Raven's tests, Wechsler tests, the Big Five Personality test, and the Perseverance Scale (see Table 2).

**Table 2:** Descriptive statistics of the main variables

	**Definition**	**N**	**Mean**	**Std. Dev**	**Min**	**Max**
**Panel A: Outcome variables**						
WISC-IV scores	WISC-IV test scores	472	88.258	10.948	59	124
Fluid Intelligence	The average of working memory, perceptual reasoning, and processing speed scores	472	8.472	1.684	3.667	13.333
Crystal intelligence	The verbal comprehension score	472	7.928	2.577	2	16
Similarities	Scores of scale	472	7.928	2.577	2	16
Digit span	Scores of scale	472	8.117	2.507	2	17
Coding	Scores of scale	472	9.71	2.481	2	19
Matrix Reasoning	Scores of scale	472	7.591	2.238	1	17
Raven's IQ	Raven's test scores	2503	93.641	17.511	5.35	133.3
Math scores of 2016	Standardized math scores	5188	.02	0.996	-2.071	2.517
Extraversion	Scores of scale	5166	3.349	0.562	1.125	5
Agreeableness	Scores of scale	5166	3.739	0.536	1.444	5
Conscientiousness	Scores of scale	5166	3.279	0.570	1.111	5
Neuroticism	Scores of scale	5166	2.913	0.625	1	5
Openness	Scores of scale	5166	3.411	0.569	1.3	4.9
**Panel B: Student Characteristics**						
Boarding	1 = Student is boarding at school	5660	0.460	0.498	0	1
Student age	A student’s age in years	5660	12.917	0.964	10	17
Student gender	1 = Male; 0 = Female	5660	0.499	0.500	0	1
Left-behind child status	1 = Father or mother migrated to a city for work for more than six months in the past year	5660	0.525	0.499	0	1
Father's education level	1 = Junior high school and above 0 = Junior high school below	5660	0.359	0.480	0	1
Mather's education level	1 = Junior high school and above 0 = Junior high school below	5660	0.213	0.410	0	1
Family asset value	Indicators of wealth in the family	5660	-0.001	1.074	-1.496	3.019
Math scores of 2015	Standardized math scores	5660	-0.112	0.974	-3.167	2.551
**Panel C:School Characteristics**						
School size	Number of students	5660	217.91	96.641	46	447
Teacher-student ratio	Number of teachers per student	5660	0.137	0.065	0.039	.474
Time required for the school to be the furthest administrative village	hours	5660	1.22	0.777	0.05	4
The rate of school boarders	The number of boarders divided by the total number of students in the school	5660	0.469	0.409	0	1
**Panel D:Teacher Characteristics**						
Teaching years	years	5660	9.072	6.954	0	38
Teacher’s gender	1 = Male; 0 = Female	5660	0.636	0.481	0	1
Teacher's education level	1 = junior college above 0 = junior college and below	5660	0.517	0.500	0	1
Teacher’s age	A teacher’s age in years	5660	32.499	5.869	22.332	56
Teacher’s title	0 = have no title;1 = third-grade title;2 = second-grade title;3 = first-grade tltle;4 = Senior professional title	5660	1.093	1.005	0	4

The data collection involved three steps: (1) recruiting and training researchers, (2) conducting questionnaire tests in schools, and (3) administering cognitive and non-cognitive ability tests. For (1), the project team recruited college students as researchers and provided uniform training and simulation exercises to ensure recruited researchers mastered standardized operations of the study, thus reducing measurement errors caused by inconsistent implementation by researchers. For (2), researchers organized students to take standardized math tests and questionnaires, which were developed by the project team in collaboration with the best secondary school teachers and calibrated to match the academic level appropriate for seventh-grade rural students. All sample schools used standardized math tests with identical questions assigned by the project team and proctored by researchers on-site. Researchers also conducted one-on-one questionnaire interviews with principals and mathematics teachers. In (3), cognitive ability tests included Raven's test and Wechsler's test. Raven's test was administered in a group and took approximately 45 min. The Wechsler test needed to be conducted one-on-one and required highly trained personnel, participants therefore received training in professional institutions. Additionally, the project team organized several practical exercises in non-sample schools to ensure the accuracy and consistency of the Wechsler test. Given the significant testing and time costs of the Wechsler test, three students from each sample class were randomly selected to take the Wechsler test individually. Students are selected based on their mathematics scores in the first research sample class, which were rank ordered into three groups: high, medium, and low; one student from each group was randomly selected for the Wechsler's test. The rest of the class took the Raven's test. The non-cognitive skills component primarily consisted of the Big Five personality test and the Perseverance Scale test, both of which were included in the student questionnaire.

### Cognitive ability

The objective of this research is to investigate students' cognitive abilities, measured using three tests: the Wechsler Intelligence Scale for Children-Fourth Edition (WISC-IV), Raven's Standard Progressive Matrices (Raven's IQ test), and a mathematics test. Cattell's (1987) suggested that cognitive abilities are divisible into two categories: crystallized intelligence and fluid intelligence [36]. The former pertains to skills attained through experience and knowledge, such as vocabulary, calculation, and verbal comprehension, whereas the latter refers to neural development, including perception, memory, and reasoning ability.

The WISC-IV is a tool for assessing intelligence in children aged 6 to 16 and comprises four indices: verbal comprehension, perceptual reasoning, working memory, and processing speed, along with the total IQ score [37]. The Chinese version of the WISC-IV short-form scale was employed in this research, which contains four subtests representing the four indices [38].^1^ The four subtests utilized for estimating the WISC IQ score were similarities, digit span, coding, and matrix reasoning. Similarities is designed to capture crystallized intelligence, while digit span, coding, and matrix reasoning are intended to measure fluid intelligence [39]. The aggregated WISC IQ score was used in the regression analysis.

The Raven's IQ test is a nonverbal test of intelligence that consists of pictorial questions related to spatial reasoning and pattern matching, which are designed to assess observational and thinking ability [40]. The test is culture-, language-, and age-neutral and consists of 60 questions that can be converted into IQ scores based on normative patterns. It is defined to capture fluid intelligence and was used for robustness testing in this study [31].^2^

The mathematics test, administered to all students in the sample, was developed by experienced secondary school mathematics teachers based on the standard high school syllabus. The test captures crystallized intelligence and was used for robustness testing in this study [33]. Several pre-studies of the questions were carried out by the research team to assess their suitability.

### Non-cognitive ability

Non-cognitive abilities represent a fundamental component of human capital and can be examined through various skills and traits, including self-control, self-esteem, self-confidence, due diligence, perseverance, self-awareness, and communication skills [45]. We employed the Big Five personality traits and the Short Grit Scales as measures of non-cognitive abilities.

DeYoung's Big Five personality traits consist of neuroticism, agreeableness, conscientiousness, extraversion, and openness, which capture diverse aspects of personality. Neuroticism assesses emotional instability and sensitivity, such as anxiety, hostility, depression, self-consciousness, impulsivity, and vulnerability. Extraversion captures interpersonal skills, positive affect, and energy levels. Openness refers to the imagination and intellectual curiosity as reflected in personal fantasy, aesthetics, feelings, actions, ideas, and values. Agreeableness evaluates how a person interacts with others through levels of trust, frankness, altruism, submissiveness, humility, and gentleness. Finally, conscientiousness assesses competence, order responsibility, effortful achievement, self-discipline, and thoughtfulness [46]. The Big Five personality traits have been widely studied and are recognized as being stable across different languages, disciplines, and raters [47, 48].

The Short Grit Scale, developed by Duckworth et al. (2007), measures perseverance and passion for long-term goals [49]. This scale consists of eight questions that evaluate student attitudes and behaviors towards long-term goals, such as the tendency to prioritize new ideas over existing plans [50]. The Short Grit Scale has demonstrated strong internal consistency, test–retest stability, and high predictive validity [51]. Grit is considered a facet of Big Five conscientiousness and has gained recent attention in the literature on human achievement. In this study, we utilized it for robustness testing.

### Model design

Consider a statistical model that links a student's cognitive and non-cognitive abilities, boarding status, and other determinants of ability as represented by:

1 $${Y}_{jis}=\alpha +{\beta }_{1}Boardin{g}_{jis}+{\beta }_{2}{W}_{jis}+{\mu }_{j}+{\varepsilon }_{jis}$$

where ${Y}_{jis}$ denotes the cognitive and non-cognitive abilities of student i in school s in county j; $Boardin{g}_{jis}$ is an indicator of the student's boarding status (1 if boarding, 0 otherwise), and ${W}_{jis}$ is a set of exogenous covariates that includes student (e.g., age and gender), family (e.g., parental education), and school (e.g., teacher qualifications and school facilities) characteristics; ${\mu }_{j}$ is county fixed effect; and the error term ${\varepsilon }_{jis}$ captures the influence of all unobserved factors.

Equation (1) may be subject to endogeneity issues for two main reasons. First, reverse causality may arise where students with lower cognitive abilities or academic performance could be more likely to choose boarding [14]. This concern is particularly true in cases such as the French excellent boarding school program, which is designed to provide elite education for disadvantaged groups. In contrast, boarding schools in rural China aim to integrate educational resources and are more likely to be chosen because of the distance between the student's home and school [26, 27, 52]. Therefore, reverse causality may not be a problem in this study. Second, omitted variables may also pose a problem, given that factors that affect students' cognitive and non-cognitive abilities may exist at multiple levels, and crucial indicators such as genetic factors and parental emotional involvement may be difficult to measure [27, 53–55].

To address these problems, we use the standard instrumental variables (IV) approach to identify an exogenous source of variation in one's boarding status. The proportion of boarders of all students in a particular school is used as an instrumental variable for boarding. This strategy is based on the assumption that the proportion of boarders is a strong predictor of one's boarding status, because a higher proportion of boarders within a school indicates a higher likelihood for students to become boarders in that school. We employ a two-stage least squares (2SLS) framework to estimate Eq. (1) and the following first-stage equation.

The first-stage equation:

2 $$Boardin{g}_{jis}={\mu }_{0}+{\mu }_{1}sc{h}_{boardingjis}+{\mu }_{2}{W}_{jis}+{\mu }_{j}+{\varepsilon }_{jis}$$

The second stage equation:

1 $${Y}_{jis}=\alpha +{\beta }_{1}Boardin{g}_{jis}+{\beta }_{2}{W}_{jis}+{\mu }_{j}+{\varepsilon }_{jis}$$

where $Boardin{g}_{jis}$ is the proportion of boarders. The definitions of other variables are the same as in Eq. (1).

## Result

### Distribution of cognitive abilities and non-cognitive abilities

**Fig. 1:**
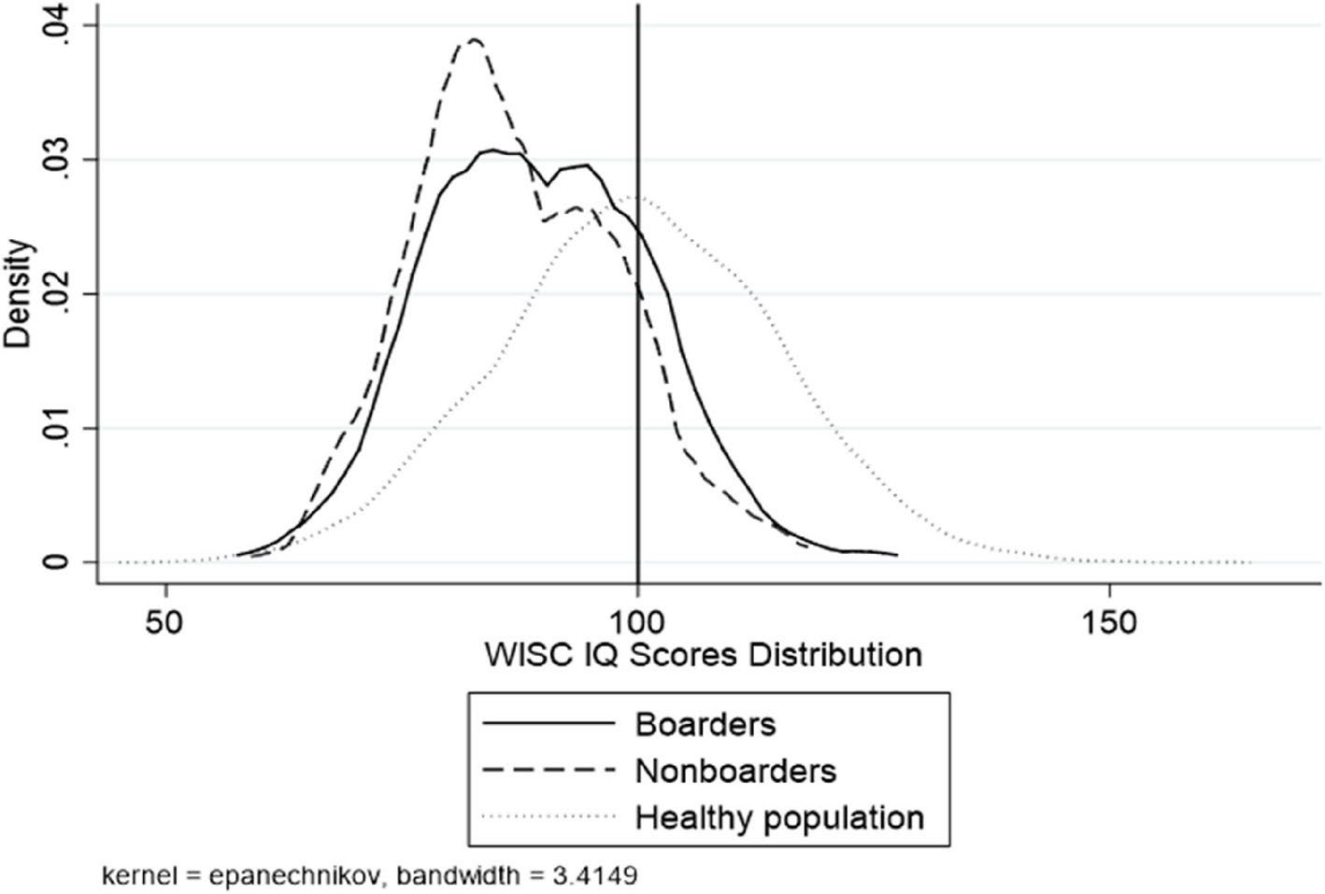
Distribution of WISC IQ scores for sample students and a healthy population. The WISC IQ scores density distribution in the healthy population is a normal distribution with a mean of 100 and a standard deviation of 15

**Fig. 2:**
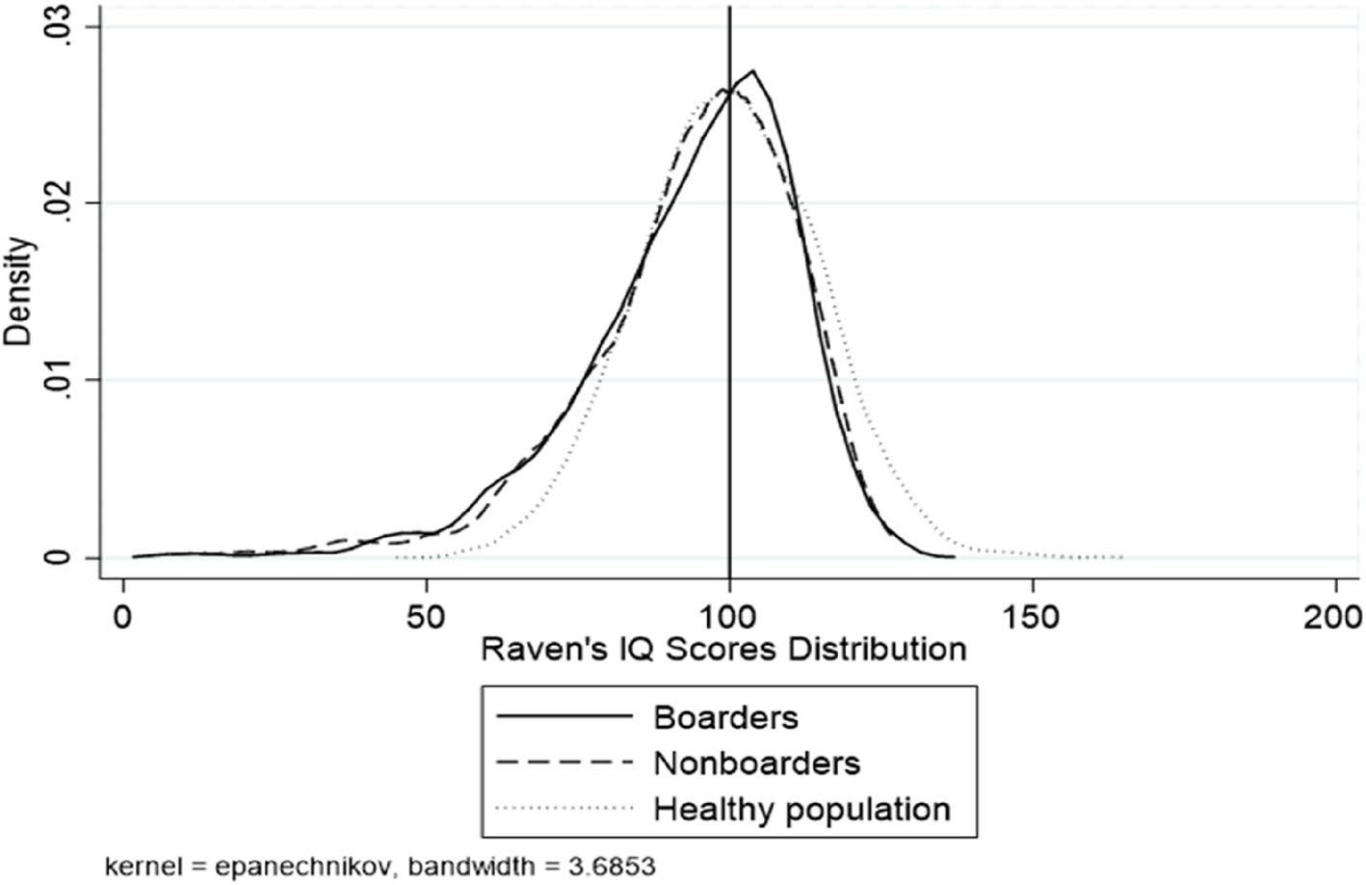
Distribution of Raven’s IQ scores for sample students and a healthy population. The Raven’s IQ scores density distribution in the healthy population is a normal distribution with a mean of 100 and a standard deviation of 15

**Fig. 3:**
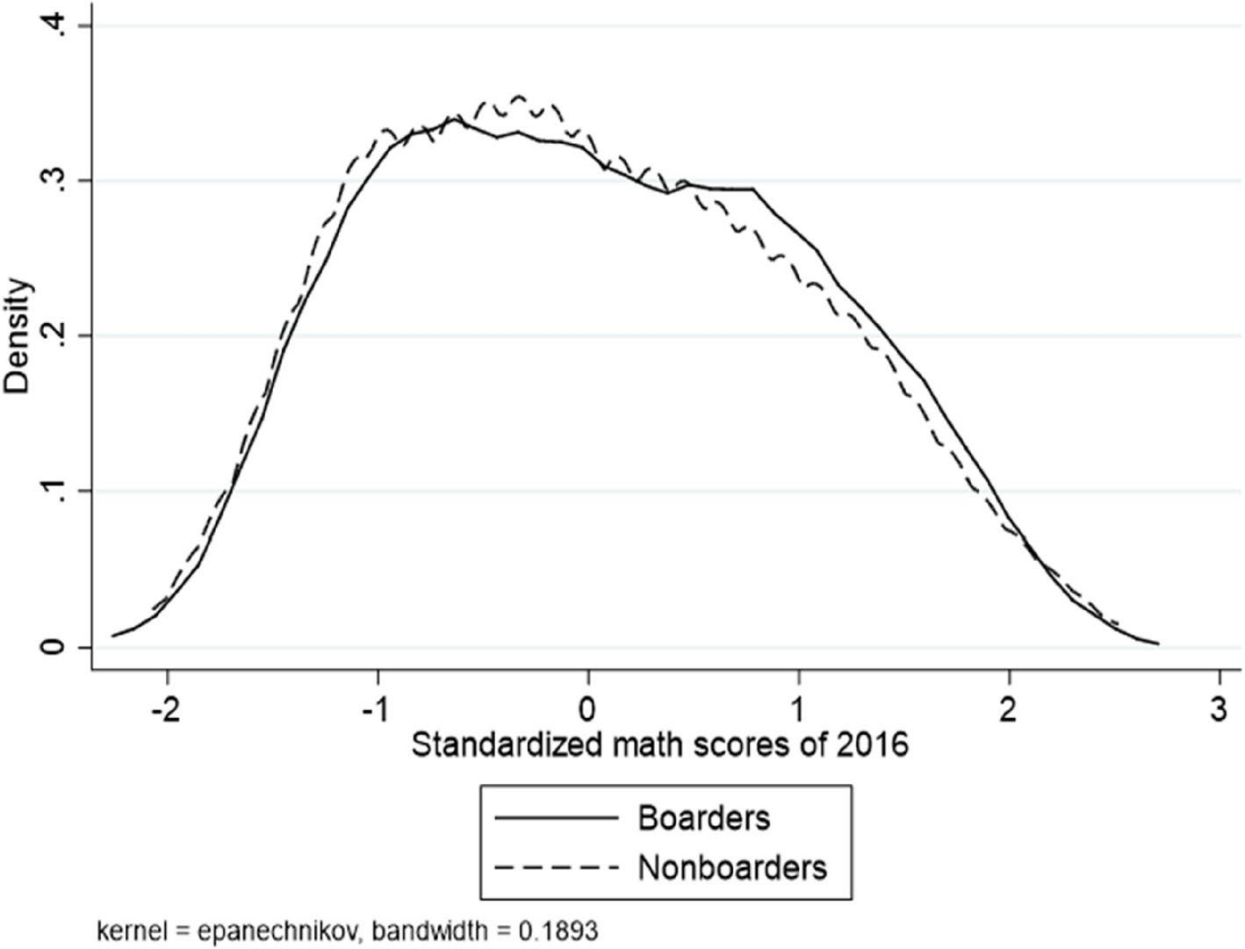
Distribution of standardized math scores for sample students. Math scores are standardized

Figure 1 shows the distribution of WISC-IV scores in our sample. The density distribution of WISC-IV scores is right-skewed for both boarding and non-boarding students compared to the norm, indicating a relatively high proportion of students with cognitive delays in our sample. Boarding students exhibit a less right-skewed distribution of WISC-IV scores compared to non-boarding students, suggesting that boarding students have higher WISC-IV scores on average. Fig. 2 shows the density distribution of Raven's IQ scores for the sample students. The estimated IQ scores on Raven's test for both boarding and non-boarding samples are not significantly different from the norm. Moreover, there is no significant difference between the Raven's IQ scores of boarding and non-boarding students. Finally, Fig. 3 illustrates the density distribution of standardized math scores for the sample students, suggesting that there is no significant difference between the boarding and non-boarding students.

Table 3 presents the differences in cognitive and non-cognitive abilities between boarding and non-boarding students. The results indicate that boarders' WISC-IV scores were 2.45 points higher than non-boarders, significant at the 5% level, and boarders' fluid intelligence scores were 0.363 points higher than non-boarder, similarly significant at the 5% level. Boarders also scored higher on the matrix reasoning scale by 0.82 points at the 1% significance level. Additionally, boarders' standardized math scores were statistically significantly higher than non-boarders. In terms of non-cognitive skills, boarders scored higher in extraversion by 0.042, but lower in agreeableness by 0.042 compared to non-boarders.

**Table 3:** Descriptive analysis of cognitive and non-cognitive abilities: comparisons between different types of students

	**Overall**		**Nonboarders**		**Boarders**		**Nonboarders—Boarders**		
	N	Mean	N	Mean	N	Mean	Difference in mean	*P*-value	Effect size (Cohen’s d)
	(1)	(2)	(3)	(4)	(5)	(6)	(7)	(8)	(9)
**Cognitive ability**									
WISC-IV scores	472	88.258	243	87.070	229	89.520	-2.450**	0.015	-0.225
Fluid Intelligence	472	8.472	243	8.296	229	8.659	-0.363**	0.019	-0.217
Crystal intelligence	472	7.928	243	7.745	229	8.122	-0.377	0.112	-0.147
Similarities	472	7.928	243	7.745	229	8.122	-0.377	0.112	-0.147
Digit span	472	8.117	243	7.984	229	8.258	-0.274	0.236	-0.109
Coding	472	9.71	243	9.712	229	9.707	0.005	0.984	0.002
Matrix Reasoning	472	7.591	243	7.193	229	8.013	-0.820***	< 0.001	-0.372
Raven's IQ	2503	93.641	1363	93.691	1140	93.582	0.109	0.877	0.006
Math scores of 2016	5188	.02	2816	-0.004	2372	0.049	-0.053*	0.055	-0.053
**Non-cognitive ability**									
Extraversion	5166	3.349	2802	3.329	2364	3.372	-0.042***	0.007	-0.076
Agreeableness	5166	3.739	2802	3.758	2364	3.716	0.042***	0.005	0.078
Conscientiousness	5166	3.279	2802	3.280	2364	3.277	0.003	0.861	0.005
Neuroticism	5166	2.913	2802	2.904	2364	2.924	-0.020	0.261	-0.031
Openness	5166	3.411	2802	3.409	2364	3.413	-0.004	0.815	-0.007
Grit scores	5166	3.349	2809	3.385	2369	3.380	0.005	0.748	0.009

Source: Author’s survey

Standard deviations in parentheses * *p* < 0.1. ** *p* < 0.05. *** *p* < 0.01

Table 4 verifies the representativeness of the WISC-IV-tested students in the sample. We examine the differences in student characteristics between those who took the test and those who did not. The results indicate no significant differences in individual characteristics, family characteristics, and baseline math scores between the two groups.

**Table 4:** Descriptive analysis of students who took the WISC-IV test: comparisons between different Characteristics of students

	**Overall**		**Students who did not take the Wechsler test**		**Students taking the Wechsler test**		**Differences**		
	N	Mean	N	Mean	N	Mean	Difference in mean	*P*-value	Effect size (Cohen’s d)
	(1)	(2)	(3)	(4)	(5)	(6)	(7)	(8)	(9)
Student age	5660	12.917	5188	12.914	472	12.948	-0.034	0.457	-0.036
Student gender	5660	.499	5188	0.499	472	0.504	-0.005	0.829	-0.010
Left-behind child status	5660	.525	5188	0.526	472	0.508	0.018	0.465	0.035
Father's education level	5660	.359	5188	0.359	472	0.360	-0.001	0.965	-0.002
Mather's education level	5660	.213	5188	0.212	472	0.225	-0.013	0.510	-0.032
Family asset value	5660	-.001	5188	0.003	472	-0.044	0.047	0.366	0.043
Math scores of 2015	5660	-.112	5188	-0.110	472	-0.139	0.029	0.529	0.030

Source: Author’s survey

Standard deviations in parentheses * *p* < 0.1. ** *p* < 0.05. *** *p* < 0.01

### Impact of boarding on cognitive abilities

Table 5 presents an analysis of the impact of boarding on cognitive abilities among rural students, specifically focusing on WISC IQ scores, fluid intelligence, and crystal intelligence. Using ordinary least squares (OLS) estimates in columns 1, 3, and 5, the results show that boarding does not have a significant effect on students' cognitive abilities. To further examine the causal relationship between boarding and cognitive abilities, two-stage least squares (2SLS) estimates are presented in columns 2, 4, and 6, and the findings indicate that boarding has no significant impact on WISC IQ scores, fluid intelligence, and crystal intelligence. The first stage regression has a high F-statistic of 41.284, indicating the exclusion of weak instrumental variables. To better understand how boarding affects students' cognitive abilities, we also estimated the impacts of boarding on the four subdimensions of WISC IQ scores, which are similarities, digit span, coding, and matrix reasoning. The results presented in Table 6. The 2SLS estimates for boarding on students’ scores in digit span has a parameter estimate of 2.024, significant at the 5% level. Since scores in digit span is a test of attention and memory, the result highlights the positive impact of boarding on students' performance in this particular cognitive dimension.

**Table 5:** Impact of boarding on students' cognitive abilities

variables	WISC IQ score	WISC IQ score	Fluid intelligence	Fluid intelligence	Crystal intelligence	Crystal intelligence
	OLS	IV	OLS	IV	OLS	IV
	(1)	(2)	(3)	(4)	(5)	(6)
Boarding	0.103	4.781	-0.048	0.635	0.181	0.934
	(1.040)	(3.592)	(0.179)	(0.573)	(0.266)	(0.925)
Student-level control variables	YES	YES	YES	YES	YES	YES
School-level control variables	YES	YES	YES	YES	YES	YES
Teacher-level control variables	YES	YES	YES	YES	YES	YES
Constant	139.817***	139.866***	16.501***	16.527***	15.396***	15.365***
	(8.633)	(8.702)	(1.408)	(1.387)	(2.309)	(2.240)
Observations	472	472	472	472	472	472
R-squared	0.418	0.393	0.371	0.348	0.286	0.286
County fixed effects	YES	YES	YES	YES	YES	YES
Phase I F-statistic values		41.284		41.284		41.284

Source: Author’s survey

Standard deviations in parentheses * *p* < 0.1. ** *p* < 0.05. *** *p* < 0.01

**Table 6:** Impact of boarding on subdimensions of cognitive abilities

variables	Similarities		Digit span		Coding		Matrix reasoning	
	OLS	IV	OLS	IV	OLS	IV	OLS	IV
	(1)	(2)	(3)	(4)	(5)	(6)	(7)	(8)
Boarding	0.181	0.934	0.278	2.024**	-0.685**	-0.472	0.263	0.352
	(0.266)	(0.925)	(0.268)	(0.982)	(0.281)	(0.892)	(0.256)	(0.831)
Student-level control variables	YES	YES	YES	YES	YES	YES	YES	YES
School-level control variables	YES	YES	YES	YES	YES	YES	YES	YES
Teacher-level control variables	YES	YES	YES	YES	YES	YES	YES	YES
Constant	15.396***	15.365***	18.264***	18.195***	19.467***	19.517***	11.771***	11.869***
	(2.309)	(2.240)	(2.216)	(2.380)	(2.098)	(2.162)	(1.976)	(2.013)
Observations	472	472	472	472	472	472	472	472
R-squared	0.286	0.274	0.201	0.134	0.272	0.271	0.223	0.223
County fixed effects	YES	YES	YES	YES	YES	YES	YES	YES
Phase I F-statistic values		41.284		41.284		41.284		41.284

Source: Author’s survey

Standard deviations in parentheses * *p* < 0.1. ** *p* < 0.05. *** *p* < 0.01

### Impact of boarding on students' non-cognitive abilities

Table 7 presents the effects of boarding on the personality traits of rural students, encompassing extraversion, agreeableness, dutifulness, neuroticism, and openness. The OLS results in columns (1), (3), (5), (7), and (9) suggest that while there is a positive relationship between boarding and the extraversion of rural students, the IV results indicate that boarding does not have a statistically significant effect on any of the five personality traits examined. Therefore, we conclude that boarding does not have any significant effects on the non-cognitive abilities of rural students.

**Table 7:** Impact of boarding on students' non-cognitive abilities

variables	Extraversion		Agreeableness		Conscientiousness		Neuroticism		Openness	
	OLS	IV	OLS	IV	OLS	IV	OLS	IV	OLS	IV
	(1)	(2)	(3)	(4)	(5)	(6)	(7)	(8)	(9)	(10)
Boarding	0.036*	-0.064	-0.034	0.023	0.009	0.109	-0.001	0.034	0.005	0.097
	(0.020)	(0.082)	(0.022)	(0.077)	(0.025)	(0.083)	(0.023)	(0.090)	(0.023)	(0.083)
Student-level control variables	YES	YES	YES	YES	YES	YES	YES	YES	YES	YES
School-level control variables	YES	YES	YES	YES	YES	YES	YES	YES	YES	YES
Teacher-level control variables	YES	YES	YES	YES	YES	YES	YES	YES	YES	YES
Constant	3.541***	3.555***	3.687***	3.707***	3.193***	3.190***	2.753***	2.747***	3.368***	3.367***
	(0.153)	(0.158)	(0.169)	(0.149)	(0.178)	(0.161)	(0.206)	(0.175)	(0.171)	(0.160)
Observations	5,166	5,166	5,166	5,166	5,166	5,166	5,166	5,166	5,166	5,166
R-squared	0.020	0.015	0.039	0.037	0.016	0.012	0.028	0.028	0.021	0.018
County fixed effects	YES	YES	YES	YES	YES	YES	YES	YES	YES	YES
Phase I F-statistic values		342.982		342.982		342.982		342.982	342.982	342.982

Source: Author’s survey

Standard deviations in parentheses * *p* < 0.1. ** *p* < 0.05. *** *p* < 0.01

### Robustness test

To enhance the robustness of the research findings, we conducted additional regression analyses. First, we added the bootstrap method to the original instrumental variables method to re-estimate the impact of boarding on students' cognitive and non-cognitive abilities. The bootstrap method involves treating the observed sample as the entire population, and repeatedly resampling with replacement from the original sample to estimate the sampling distribution. This approach can provide an estimate of the distribution without introducing bias. In this paper, we conducted 1000 bootstrap samples and then used the instrumental variables method for estimation, which can provide more robust standard errors. Tables 8 and 9 show the results, which indicate that boarding still has a significant positive effect at the 10% level on students' scores in digit span and no significant effect on students' non-cognitive abilities, which is consistent with the results above.

**Table 8:** Robustness test I: bootstrap sampling—cognitive abilities

variables	WISC IQ score	Fluid intelligence	Crystal intelligence	Similarities	Digit span	Coding	Matrix reasoning
	(1)	(2)	(3)	(4)	(5)	(6)	(7)
Boarding	4.781	0.635	0.898	0.934	2.024*	-0.472	0.352
	(3.725)	(0.621)	(0.877)	(1.022)	(1.034)	(0 .990)	(0.890)
Student-level control variables	YES	YES	YES	YES	YES	YES	YES
School-level control variables	YES	YES	YES	YES	YES	YES	YES
Teacher-level control variables	YES	YES	YES	YES	YES	YES	YES
Constant	139.866***	16.527***	15.373***	15.365***	18.195***	19.517***	11.869***
	(9.140)	(1.550)	(2.439)	(2.520)	(2.340)	(2.289)	(2.113)
Observations	472	472	472	472	472	472	472
R-squared	0.393	0.348	0.274	0.274	0.133	0.271	0.223
County fixed effects	YES	YES	YES	YES	YES	YES	YES
Phase I F-statistic values	41.732	41.732	41.732	41.732	41.732	41.732	41.732

Source: Author’s survey

Standard deviations in parentheses * *p* < 0.1. ** *p* < 0.05. *** *p* < 0.01

**Table 9:** Robustness test I: bootstrap sampling-non-cognitive abilities

variable	Extraversion	Agreeableness	Conscientiousness	Neuroticism	Openness
	(1)	(2)	(3)	(4)	(5)
Boarding	-0.064	0.023	0.109	0.034	0.097
	(0.087)	(0.076)	(0.088)	(0.092)	(0.080)
Student-level control variables	YES	YES	YES	YES	YES
School-level control variables	YES	YES	YES	YES	YES
Teacher-level control variables	YES	YES	YES	YES	YES
Constant	3.555***	3.707***	3.190***	2.747***	3.367***
	(0.156)	(0.148)	(0.161)	(0.176)	(0.162)
Observations	5,166	5,166	5,166	5,166	5,166
R-squared	0.015	0.037	0.012	0.028	0.018
County fixed effects	YES	YES	YES	YES	YES
Phase I F-statistic values	342.982	342.982	342.982	342.982	342.982

Source: Author’s survey

Standard deviations in parentheses * *p* < 0.1. ** *p* < 0.05. *** *p* < 0.01

Second, we performed robustness tests using Raven's IQ, standardized math, and grit scores as additional measures of cognitive and non-cognitive abilities. Raven's IQ and standardized math scores are measures of fluid and crystal intelligence, respectively. The results in Table 10 suggest that boarding does not have a significant effect on students' Raven's IQ and standardized math scores, which are consistent with previous findings. Grit is closely related to conscientiousness in the Big Five personality traits, and although the two are not identical, they share strong similarities such as diligence and perseverance [56]. It has also been shown that grit is a more refined measure of conscientiousness [57, 58]. However, columns (5) and (6) of Table 10 show that boarding does not have a significant effect on students' grit scores, which are consistent with the previous results.

**Table 10:** Robustness test II: impact of boarding on students' cognitive and non-cognitive abilities

variables	Raven’s IQ scores		Standardization Math scores of 2016		Grit scores	
	(1)	(2)	(3)	(4)	(5)	(6)
Boarding	-0.548	1.669	-0.052	0.086	0.003	-0.014
	(0.855)	(2.933)	(0.039)	(0.110)	(0.021)	(0.083)
Student-level control variables	YES	YES	YES	YES	YES	YES
School-level control variables	YES	YES	YES	YES	YES	YES
Teacher-level control variables	YES	YES	YES	YES	YES	YES
Constant	167.539***	170.819***	2.049***	2.226***	3.146***	3.209***
	(7.639)	(5.789)	(0.330)	(0.214)	(0.153)	(0.161)
Observations	2,503	2,503	5,188	5,188	5,188	5,188
R-squared	0.337	0.335	0.426	0.423	0.426	0.423
County fixed effects	YES	YES	YES	YES	YES	YES
Phase I F-statistic values		172.725		348.218		349.966

Source: Author’s survey

Standard deviations in parentheses * *p* < 0.1. ** *p* < 0.05. *** *p* < 0.01

### Heterogeneity analysis

In addition to analyzing the effects of boarding, we investigate the heterogeneity of these effects along three dimensions: gender, whether the student is a left-behind child, and family asset status. Family asset status is defined by ranking students' family asset indices from smallest to largest, and students in the top 25% of the sample are classified as having bad family conditions, with a dummy variable indicating whether a student's family conditions are bad. Table 11 presents the results of the heterogeneity analysis for cognitive abilities. The estimates in Panels A and B show that boarding has a significant positive effect on boys' WISC IQ scores, particularly in fluid intelligence, as evidenced by improvements in digit span and matrix reasoning. These findings suggest that boarding enhances boys' memory, attention, and reasoning abilities. Panels C and D indicate that boarding also has a significant positive impact on left-behind children's WISC IQ scores, again largely reflected in fluid intelligence. Boarding increases the digit span score (memory and attention) of left-behind children by 2.952 points (p < 0.05). However, the coding score for non-left-behind children is negatively affected, indicating that boarding reduces their transcription speed, accuracy, general learning ability, and anti-distraction ability. Panels E and F demonstrate that boarding has a significant positive effect at the 5% level on the digit span scores (memory and attention) of students with good family conditions, while there is no significant effect on students' cognitive abilities from low-income families.

**Table 11:** Results of the heterogeneity analysis of the impact of boarding on students' cognitive abilities

variables	WISC IQ score		Fluid intelligence		Crystal intelligence		Similarities		Digit span		Coding		Matrix reasoning	
	OLS	IV	OLS	IV	OLS	IV	OLS	IV	OLS	IV	OLS	IV	OLS	IV
	(1)	(2)	(3)	(4)	(5)	(6)	(7)	(8)	(9)	(10)	(11)	(12)	(13)	(14)
**Panel A: Sample of male students**														
Boarding	0.448	9.128**	0.132	1.494**	-0.205	0.998	-0.205	0.998	0.346	2.756**	-0.604	0.295	0.654*	1.432*
	(1.418)	(3.991)	(0.250)	(0.609)	(0.362)	(1.046)	(0.362)	(1.046)	(0.422)	(1.110)	(0.399)	(0.918)	(0.378)	(0.867)
Observations	238	238	238	238	238	238	238	238	238	238	238	238	238	238
Phase I F-statistic values		36.872		36.872		36.872		36.872		36.872		36.872		36.872
**Panel B: Sample of female students**														
Boarding	-0.584	-1.371	-0.336	-0.275	0.512	0.167	0.512	0.167	-0.102	1.284	-0.674	-0.456	-0.231	-1.652
	(1.764)	(7.339)	(0.299)	(1.193)	(0.404)	(1.796)	(0.404)	(1.796)	(0.427)	(1.934)	(0.431)	(1.831)	(0.402)	(1.771)
Observations	234	234	234	234	234	234	234	234	234	234	234	234	234	234
Phase I F-statistic values		9.784		9.784		9.784		9.784		9.784		9.784		9.784
**Panel C: Sample of left-behind children**														
Boarding	-0.140	9.751*	0.116	1.652**	-0.458	1.006	-0.458	1.006	0.671	2.952**	-0.008	1.855	-0.315	0.147
	(1.653)	(5.256)	(0.266)	(0.828)	(0.438)	(1.344)	(0.438)	(1.344)	(0.425)	(1.385)	(0.434)	(1.255)	(0.354)	(1.159)
Observations	240	240	240	240	240	240	240	240	240	240	240	240	240	240
Phase I F-statistic values		21.891		21.891		21.891		21.891		21.891		21.891		21.891
**Panel D: Sample of non-left-behind children**														
Boarding	1.909	-1.566	0.045	-0.770	0.925***	1.363	0.925***	1.363	0.079	1.470	-0.623	-3.446**	0.680*	-0.336
	(1.305)	(5.544)	(0.243)	(0.913)	(0.338)	(1.436)	(0.338)	(1.436)	(0.389)	(1.535)	(0.383)	(1.551)	(0.353)	(1.311)
Observations	232	232	232	232	232	232	232	232	232	232	232	232	232	232
Phase I F-statistic values		15.299		15.299		15.299		15.299		15.299		15.299		15.299
**Panel E: Sample with poor family conditions**														
Boarding	-1.441	6.109	-0.280	0.809	-0.012	1.311	-0.012	1.311	-0.255	1.519	-1.246**	0.064	0.661	0.842
	(1.942)	(5.490)	(0.311)	(0.863)	(0.465)	(1.329)	(0.465)	(1.329)	(0.527)	(1.543)	(0.587)	(1.327)	(0.596)	(1.288)
Observations	144	144	144	144	144	144	144	144	144	144	144	144	144	144
Phase I F-statistic values		15.818		15.818		15.818		15.818		15.818		15.818		15.818
**Panel F: Sample with good family conditions**														
Boarding	0.940	7.132	0.085	1.257	0.283	0.630	0.283	0.630	0.530	2.884**	-0.385	0.197	0.111	0.690
	(1.365)	(5.126)	(0.233)	(0.831)	(0.324)	(1.304)	(0.324)	(1.304)	(0.366)	(1.402)	(0.346)	(1.282)	(0.318)	(1.174)
Observations	328	328	328	328	328	328	328	328	328	328	328	328	328	328
Phase I F-statistic values		19.995		19.995		19.995		19.995		19.995		19.995		19.995

Source: Author’s survey

Standard deviations in parentheses * *p* < 0.1. ** *p* < 0.05. *** *p* < 0.01

The heterogeneity analysis of non-cognitive abilities is presented in Table 12. Panels A and B show gender differences in the effect of boarding on students' non-cognitive abilities. Specifically, there is a significant positive effect of boarding on girls' conscientiousness at the 10% statistical level, indicating that boarding enhances girls' abilities in areas such as responsibility, workability, and self-control. However, no significant differences were found in the effect of boarding on non-cognitive abilities in other aspects. These findings suggest that boarding schools have varying effects on different dimensions of students' cognitive and non-cognitive abilities, highlighting the importance of considering heterogeneity in understanding the overall impact of boarding school education.

**Table 12:** Results of the heterogeneity analysis of the effect of boarding on students' non-cognitive abilities

Variables	Extraversion		Agreeableness		Conscientiousness		Neuroticism		Openness	
	OLS	IV	OLS	IV	OLS	IV	OLS	IV	OLS	IV
	(1)	(2)	(3)	(4)	(5)	(6)	(7)	(8)	(9)	(10)
**Panel A: Sample of male students**										
Boarding	0.002	-0.113	-0.060*	-0.099	0.025	0.006	0.023	0.053	0.031	0.101
	(0.027)	(0.111)	(0.031)	(0.112)	(0.036)	(0.117)	(0.032)	(0.125)	(0.032)	(0.115)
Observations	2,578	2,578	2,578	2,578	2,578	2,578	2,578	2,578	2,578	2,578
Phase I F-statistic values		169.575		169.575		169.575		169.575		169.575
**Panel B: Sample of female students**										
Boarding	0.074**	0.006	-0.014	0.130	-0.016	0.222*	-0.027	0.002	-0.029	0.112
	(0.033)	(0.119)	(0.028)	(0.106)	(0.031)	(0.118)	(0.033)	(0.130)	(0.030)	(0.118)
Observations	2,588	2,588	2,588	2,588	2,588	2,588	2,588	2,588	2,588	2,588
Phase I F-statistic values		176.668		176.668		176.668		176.668		176.668
**Panel C: Sample of left-behind children**										
Boarding	0.032	-0.099	-0.025	0.003	0.033	0.121	0.000	0.079	0.023	0.035
	(0.025)	(0.126)	(0.027)	(0.115)	(0.033)	(0.126)	(0.035)	(0.136)	(0.030)	(0.123)
Observations	2,715	2,715	2,715	2,715	2,715	2,715	2,715	2,715	2,715	2,715
Phase I F-statistic values		142.219		142.219		142.219		142.219		142.219
**Panel D: Sample of non-left-behind children**										
Boarding	0.037	-0.084	-0.036	0.050	-0.022	0.052	-0.020	0.008	-0.009	0.179
	(0.032)	(0.117)	(0.032)	(0.115)	(0.035)	(0.121)	(0.033)	(0.133)	(0.034)	(0.123)
Observations	2,451	2,451	2,451	2,451	2,451	2,451	2,451	2,451	2,451	2,451
Phase I F-statistic values		172.425		172.425		172.425		172.425		172.425
**Panel E: Sample with poor family conditions**										
Boarding	0.025	-0.157	-0.039	-0.056	0.019	0.173	0.006	0.129	-0.007	0.135
	(0.034)	(0.155)	(0.041)	(0.146)	(0.042)	(0.159)	(0.038)	(0.176)	(0.038)	(0.164)
Observations	1,399	1,399	1,399	1,399	1,399	1,399	1,399	1,399	1,399	1,399
Phase I F-statistic values		84.057		84.057		84.057		84.057		84.057
**Panel F: Sample with good family conditions**										
Boarding	0.044*	-0.016	-0.030	0.065	0.003	0.081	-0.005	-0.002	0.005	0.063
	(0.025)	(0.103)	(0.025)	(0.097)	(0.028)	(0.104)	(0.028)	(0.113)	(0.025)	(0.102)
Observations	3,767	3,767	3,767	3,767	3,767	3,767	3,767	3,767	3,767	3,767
Phase I F-statistic values		225.648		225.648		225.648		225.648		225.648

Source: Author’s survey

Standard deviations in parentheses * *p* < 0.1. ** *p* < 0.05. *** *p* < 0.01

## Conclusion and discussion

This study examines the impact of boarding on the cognitive and non-cognitive abilities of 5,660 junior high school students from 160 schools in rural northwest China using an instrumental variable (IV) approach. Our findings suggest that boarding has a significant positive effect on the digit span scores of junior high school students, which is a key component of the Working Memory Index in WISC, and suggests that boarding improves students’ memory and attention. However, we did not find any significant effects on other aspects of cognitive ability, such as logical thinking, reasoning, and transcription speed and accuracy. Additionally, we found no effects on non-cognitive abilities. To ensure the robustness of our findings, we conducted bootstrap and alternative variable tests, which supported our main results.

We used comprehensive indicators of cognitive abilities, namely crystal and fluid intelligence, to investigate the impact of boarding on rural students' cognitive abilities. Our analysis shows that boarding has a significant impact only on the ability of students to perform digit-span tasks, which is an important component of the Working Memory Index in the WISC. This finding suggests that boarding can positively affect students' memory and attention skills. One possible explanation for this result is that intensive and continuous learning or training can enhance an individual's cognitive abilities [59–61]. Moreover, boarders have a more collaborative learning and living environment in comparison to commuters, and they spend more time on general studies and homework [29]. Thus, boarding can significantly improve students' cognitive abilities. Furthermore, previous research has shown that the breadth of children's working memory increases linearly between the ages of 4 and 15 [62]. As boarders spend more time at school than commuters, they may have more opportunities to exercise their cognitive abilities, particularly in the area of attention and memory. Therefore, the positive effect of boarding on their cognitive abilities is likely to be reflected in their performance on digit span tasks. Most previous studies on the relationship between boarding and students' cognitive ability have often used academic performance (such as grades in math, language, and reading) as a proxy variable for cognitive ability. The conclusions drawn from these studies have been mixed. For instance, some studies suggest a positive effect of boarding on students' math performance in elementary school [19, 23], while others demonstrate a significant negative influence on reading scores [26, 28]. On the other hand, boarding has been linked to a positive effect on math and language scores among junior high school students [29, 63]. Nonetheless, academic performance merely measures a certain dimension of students' cognitive ability, often reflecting crystallized intelligence and not offering a comprehensive evaluation of cognitive ability. From this perspective, this study employs the Wechsler test to more precisely gauge the impact of boarding on students' cognitive ability. In the existing literature, China Education Tracking Survey (CEPS) data is the only source that gauged students' cognitive ability through a unified scale (but not the Wechsler and Raven tests utilized in this study). Their findings indicated that boarding does not significantly impact students' cognitive abilities [63], which broadly aligns with the results of our study.

Moreover, regarding boarding and noncognitive ability, the current literature has primarily focused on mental health indicators as measures of noncognitive ability (e.g., depression, bullying, etc.). For instance, studies have pointed to potential psychological risks associated with boarding for students [27, 28, 64–66]. However, the Big Five personality traits measurement is an internationally recognized and widely employed tool for non-cognitive abilities. Notably, there is a scarcity of literature investigates the impact of boarding on students' non-cognitive skills within the framework of these five dimensions.

We conducted further analysis on the heterogeneity of the effects of boarding on the cognitive and non-cognitive abilities of rural junior high school students by gender, stay-at-home status, and family conditions. Our results indicate that the effect of boarding on the cognitive abilities of boys is greater than that of girls, particularly in fluid intelligence, as measured by digit span and matrix reasoning scales that show improved memory, attention, and reasoning abilities. This could be due to differences in time management skills between boys and girls, with boys benefitting from the external discipline and communal learning atmosphere in boarding schools [67]. For left-behind students, boarding has a greater impact on their cognitive abilities, particularly in the areas of memory and attention, as they lack parental engagement and receive more support from teachers [13]. Additionally, boarding can have a positive impact on the cognitive abilities of students from better-off families due to improved nutritional intake in school, which is associated with better cognitive development [68]. Existing research often uses academic performance as a proxy for cognitive abilities, which may not accurately capture the full range of cognitive skills. Furthermore, the positive effects of boarding on non-cognitive abilities were not observed in our study, suggesting the need for further investigation into the impact of boarding on non-cognitive development.

Our analysis of the heterogeneous effects of boarding on students' cognitive abilities also extends to their non-cognitive abilities. We find that boarding has a more significant impact on girls' conscientiousness of the Big Five personality traits, which is consistent with previous research on gender differences in conscientiousness [69]. This may be attributed to female personality traits and the role of gender as a moderating variable affecting individual commitment [70]. Social role theory also suggests that individuals of different genders develop different senses of group identity, causing them to behave differently in different social situations [71]. Therefore, greater commitment by girls to group characteristics may explain why they are more likely to be influenced by the communal learning atmosphere and external discipline that boarding schools provide. Interestingly, we did not find a significant effect of boarding on non-cognitive abilities among left-behind children or those from different family backgrounds. This finding supports our main estimation results and suggests that boarding may not have a substantial impact on the non-cognitive abilities of rural middle school students. Furthermore, we ruled out the possibility of positive and negative effects of boarding in different subsamples cancelling each other out, which strengthens this conclusion.

In conclusion, our study provides evidence that boarding schools is not detrimental to the development of new human capital, including cognitive and non-cognitive abilities, among rural junior high school students. Moreover, boarding positively affects some dimensions of students' cognitive abilities, particularly in the areas of memory and attention, and has a greater effect on certain subgroups such as boys, left-behind children, and students from better-off families. The growing prevalence of boarding schools in rural areas underscores the need to explore ways to further enhance students' cognitive skills and foster the development of non-cognitive or socio-emotional abilities in their daily boarding life. This issue deserves ongoing attention and efforts from educators and policymakers.

We do acknowledge one limitation of this study. While our analysis and findings indicate that boarding has not significantly impacted the cognitive and non-cognitive abilities of rural junior high school students, it remains possible that potential adverse effects are still present. It’s worth noting that the cognitive and non-cognitive skills evaluated in this paper using measurement scales may not fully encompass the nuances of various students’ behaviors tied to boarding. For instance, aspects like the dynamics between boarders and roommates, as well as the prolonged separation of boarders from parents, could potentially exert negative influences on their non-cognitive abilities, including interpersonal skills and emotional well-being [20, 21, 63, 72]. This points to both the limitation of our current study and the necessity for further research.

## Data Availability

The datasets used and analyzed during the current study are available from the corresponding author on reasonable request. **Availability of methods** All methods were carried out in accordance with relevant guidelines and regulations.

## Footnotes

1 WISC-IV is a clinical instrument created by David Wechsler in the United States. It is utilized to individually evaluate the intelligence of children between the aged 6 and 16 years and is internationally recognized as one of the most authoritative and valid personal intelligence tests. It was published in 1949 and updated to the fourth edition (WISC-IV) in 2003. In 2007, Prof. Houcan Zhang of the School of Psychology at Beijing Normal University, introduced the fourth edition to China. Leveraging Chinese census data, 1100 people were proportionally sampled from 12 provinces in mainland China, with 100 people in each age group, to establish the Chinese version of the norm (with a mean of 100 and a standard deviation of 15). Resulting in a calculated mean of 88.258 and a standard deviation of 10.948 for the students in our study’s samples. The average reliability coefficients of each synthetic score of the scale ranged from 0.87 to 0.97, meeting the standards set by the original American version. For this paper, the results of the Wechsler test were adjusted according to the Chinese norm [38].

2 Raven's Standard Progressive Matrices is designed by the British psychologist Raven in 1938 designed for people over age of 5.5 with normal intellectual development. The norms used in this paper are from the 1989 version (a sample of 5,108 students aged between 5 and 70 underwent testing to determine China’s Raven’s norm for the Raven test, with a mean of 100 and a standard deviation of 15) [41], as no updated norms have been established specifically for the Chinese population. Related studies in China have also used this test [42–44]. The split-half and retest reliabilities of the Raven test were 0.95 (p < 0.1) for the single-sample test and 0.82 (p < 0.1) for the retest. Concurrent validity was 0.71 (P < 0.1), and predictive validity was 0.45 (P < 0.1).
